# Apolipoprotein E isoform dependently affects Tat-mediated HIV-1 LTR transactivation

**DOI:** 10.1186/s12974-018-1129-1

**Published:** 2018-03-20

**Authors:** Nabab Khan, Gaurav Datta, Jonathan D. Geiger, Xuesong Chen

**Affiliations:** Department of Biomedical Sciences, University of North Dakota School of Medicine and Health Sciences, 504 Hamline Street, Grand Forks, ND 58203 USA

**Keywords:** ApoE, HIV-1 Tat, Low-density lipoprotein receptor-related protein 1, ApoE mimetic peptide, HIV-1 LTR transactivation

## Abstract

**Background:**

Apolipoprotein E (ApoE) is the major carrier protein that mediates the transport and delivery of cholesterol and other lipids in the brain. Three isoforms of ApoE (ApoE2, ApoE3, ApoE4) exist in humans, and their relative expression levels impact HIV-1 infection, HIV-1/AIDS disease progression, and cognitive decline associated with HIV-1-associated neurocognitive disorder. Because HIV-1 Tat, a viral protein essential for HIV-1 replication, can bind to low-density lipoprotein receptor-related protein 1 (LRP1) that controls ApoE uptake in the brain, we determined the extent to which different isoforms of ApoE affected Tat-mediated HIV-1 LTR transactivation.

**Methods:**

Using U87MG glioblastoma cells expressing LTR-driven luciferase, we determined the extent to which LRP1 as well as ApoE2, ApoE3, and ApoE4 affected Tat-mediated HIV-1 LTR transactivation.

**Results:**

A specific LRP1 antagonist and siRNA knockdown of LRP1 both restricted significantly Tat-mediated LTR transactivation. Of the three ApoEs, ApoE4 was the least potent and effective at preventing HIV-1 Tat internalization and at decreasing Tat-mediated HIV-1 LTR transactivation. Further, Tat-mediated LTR transactivation was attenuated by an ApoE mimetic peptide, and ApoE4-induced restriction of Tat-mediated LTR transactivation was potentiated by an ApoE4 structure modulator that changes ApoE4 into an ApoE3-like phenotype.

**Conclusions:**

These findings help explain observed differential effects of ApoEs on HIV-1 infectivity and the prevalence of HAND in people living with HIV-1 infection and suggest that ApoE mimetic peptides and ApoE4 structure modulator might be used as a therapeutic strategy against HIV-1 infection and associated neurocognitive disorders.

**Electronic supplementary material:**

The online version of this article (10.1186/s12974-018-1129-1) contains supplementary material, which is available to authorized users.

## Background

Apolipoprotein E (ApoE), an apolipoprotein that mediates the transport and delivery of cholesterol and lipids through cell surface ApoE receptors [1], is highly expressed in the brain, and it plays a critical role in maintaining brain function [2, 3]. Three isoforms of ApoE with amino acid differences restricted to residues 112 and 158 have been identified in humans [1], and these differences affect the structure of ApoE isoforms, influence their ability to bind lipids and receptors [4–6], and contribute to disease pathogenesis. ApoE4, in addition to its involvement in Alzheimer’s disease [7–10] and cerebrovascular disease [11], is known to enhance HIV-1 infection [12], accelerate HIV-1 disease progression [12, 13], and increase the incidence of HIV-1-associated neurocognitive disorders (HAND) [14, 15].

Affecting 37 million people worldwide, HIV-1 virus can enter the brain within the first week of systemic infection [16, 17] after which it can harbor in CSF, perivascular macrophages, microglia, and astrocytes [18]. Although combined anti-retroviral therapy suppresses almost completely HIV-1 replication in plasma and CSF, reservoirs of HIV-1 exist and chronic low levels of neuroinflammation persist and appear to result in the 50% prevalence rate of HAND [19, 20]. Thus, additional strategies are needed to block viral reactivation to maintain HIV-1 in a state of prolonged silencing, to prevent disease progression, and to prevent HAND. One strategy might be the prevention of the transactivation of transcription protein (Tat) from activating HIV-1 replication because in the absence of Tat, transcription terminates prematurely due to inefficient elongation of the long terminal repeat (LTR) [21–23].

Secreted from HIV-1-infected or HIV-transfected cells [24], Tat enters all CNS cells by endocytosis with the assistance of different receptors including low-density lipoprotein receptor-related protein 1 (LRP1), the major receptor for ApoE uptake in the brain; consequently, Tat is considered a key virulent factor of HIV-1 enhancing HIV infectivity and contributing to HIV/AIDS disease progression and the development of HAND [25–27]. Further, of the three known human isoforms of ApoE, ApoE4 enhances HIV-1 infection and accelerates HIV-1 disease progression [12, 13, 28]. Accordingly, here, we tested the hypothesis that ApoE isoform dependently affects Tat-mediated HIV-1 LTR transactivation. Principally, we found that LRP1 was involved in Tat-mediated HIV-1 LTR transactivation, that ApoE4 was less effective than ApoE2 and ApoE3 in preventing HIV-1 Tat internalization and Tat-mediated HIV-1 LTR transactivation, that an ApoE mimetic peptide attenuated Tat-mediated LTR transactivation, and that an ApoE4 structure modulator enhanced the effectiveness of ApoE4 in blocking Tat-mediated HIV-1 LTR transactivation. These findings contribute to our understanding of ApoE and HIV-1 and suggest that ApoE mimetic peptides might be a therapeutic strategy against HIV-1 and HAND.

## Methods

### Cell culture

U87MG glioblastoma cells were cultured in 1× DMEM (Invitrogen) supplemented with 10% fetal calf serum and penicillin/streptomycin (Invitrogen). U87MG cells were stably transfected with luciferase reporter gene under the control of HIV-1 LTR promoter following neomycin (Sigma-Aldrich) selection pressure. These cells were provided generously by Dr. Lena Al-Harthi (Rush University, Chicago). ApoE2 and ApoE3 were purchased from Sigma-Aldrich, and ApoE4 from Abcam. Human plasma HDL were obtained from Kalen Biomedical and ApoE-deprived HDL3 (*d* = 1.125–1.21 g/ml) were prepared [29].

### Luciferase reporter assay for Tat-mediated HIV-1 LTR transactivation

U87MG cells were seeded at a confluency level of about 50% (10,000 cells) on 96 well plates 1 day prior to being taken for experimentation. Cells were incubated with ApoE isoforms with or without HDL in the absence and presence of 2 μg/ml HIV-1 Tat (ABL Inc. and NIH AIDS program) protein and chloroquine (100 μM). Forty-eight hours post-incubation, luciferase activity assays (Promega) were performed and relative luminescence was quantified using a fluorometer/luminometer plate reader (Spectra MAX GEMINI EM). Similar protocols were followed using cells treated with HDL, LRP1 receptor-associated protein (RAP from American Research products), ApoE mimetic peptide (H-Gly-Arg-Leu-Val-Gln-Tyr-Arg-Gly-Glu-Val-Gln-Ala-Met-Leu-Gly-Gln-Ser-Thr-Glu-Glu-Leu-Arg-Val-Arg-Leu-Ala-Ser-His-Leu-Arg-Lys-Leu-Arg-Lys-Arg-Leu-Leu-Arg-Asp-OH from Anaspac Inc.), or ApoE4 structure corrector (PH002, Calbiochem).

### RNA interference

U87MG cells were transfected with negative control siRNA (Ambion) and specific targeted LRP1 siRNA (sense: CAGACAAGAUUGAACGGAUtt and anti-sense: AUCCGUUCAAUCUUGUCUGTc from Ambion) by Jetprime reagent (Polyplus). Twenty-four hours post-transfection, cells were treated with HIV-1 Tat protein in the presence of chloroquine (100 μM) for 48 h and then taken for luciferase assay. Transfection efficiency was determined by protein levels of LRP1.

### Immunoblotting

Cells were harvested and lysed in 1× RIPA lysis buffer (Thermofisher) plus 10 mM NaF, 1 mM Na_3_VO_4_, and Protease Inhibitor Cocktail (Sigma). After centrifugation (14,000×*g* for 10 min at 4 °C), supernatants were collected, and protein concentrations were determined with a DC protein assay (Bio-Rad). Proteins (10 μg) were separated by SDS-PAGE (12% gel) and transferred to polyvinylidene difluoride membranes (Millipore). The membranes were incubated overnight at 4 °C with antibodies against GAPDH (Abcam) and LRP1 (Abcam). The blots were developed with enhanced chemiluminescence, and bands were visualized and analyzed by our LI-COR Odyssey Fc Imaging System.

### HIV-1 Tat internalization assay

Quantitative analysis of HIV-1 Tat internalization was performed using a method as described previously, but with minor modifications [30]. Cells plated on glass-bottom 35-mm^2^ tissue culture dishes were co-incubated with ApoE2, ApoE3, or ApoE4 (5,10 μg/ml) and FITC-labeled HIV-1 Tat (5 μg/ml; purchased from Rockland) for 90 min at 37 °C in the presence of chloroquine (100 μM). Cells were washed with an acid wash solution (0.2 M acetic acid, 0.5 M NaCl, pH 2.8) at 4 °C for 10 min and then washed with ice-cold PBS for 5 min to remove surface-bound HIV-1 Tat-FITC. Cells were fixed in 4% paraformaldehyde, and images were taken with a confocal laser-scanning microscope (Zeiss LSM800). All experiments were performed in triplicate. The average integrated intensity of HIV-1 Tat-FITC green signal per cell was calculated for each dish using ImageJ software.

### Statistical analysis

All data were expressed as means ± SD. Statistical significance between two groups was analyzed with a Student’s *t* test, and statistical significance among multiple groups was analyzed with one-way ANOVA plus a Tukey post hoc test. *p* < 0.05 was considered to be statistically significant.

## Results

### LRP1 is involved in Tat-mediated HIV-1 LTR transactivation

**Fig. 1:**
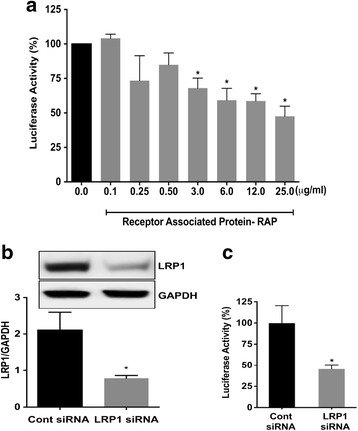
Involvement of LRP1 in Tat-mediated HIV-1 LTR transactivation. **a** The LRP1 antagonist, receptor-associated protein (RAP), concentration dependently reduced Tat-mediated HIV-1 LTR transactivation (*n* = 3; *p < 0.05). **b** Protein expression levels of LRP1 were significantly (*n* = 3, *p* < 0.05) reduced in U87MG cells transfected with LRP1 siRNA (100 nM) compared to cells transfected with control (scrambled) siRNA. **c** siRNA knockdown of LRP1 reduced significantly (*n* = 3; *p < 0.05) Tat-mediated HIV-1 LTR transactivation

For our studies, we measured Tat-mediated HIV-1 LTR transactivation using U87MG glioblastoma cells stably transfected with HIV-1 LTR-driven luciferase. With this system, concentration-dependent increases in Tat-mediated HIV-1 LTR transactivation were observed when exogenous HIV-1 Tat protein was co-applied at an optimal concentration of 100 μM chloroquine (Additional file 1: Figure S1), a weak base that neutralizes the acidic environment of lysosomes, prevents HIV-1 Tat degradation, and enhances nuclear delivery of HIV-1 Tat [26, 31–33]. Thus, many factors related to HIV-1 infection could affect Tat-mediated LTR transactivation. Given that gp120 and TNFα have both been shown to neutralize endolysosome pH [34–36], we determined the extent to which gp120 and TNFα affected Tat-mediated LTR transactivation in the absence of chloroquine, and neither affected Tat-mediated LTR transactivation (Additional file 1: Figure S2). However, we demonstrated that other endolysosome de-acidifying reagents acted in a similar way as chloroquine to enhance Tat-mediated LTR transactivation (Additional file 1: Figure S2), and these reagents include LYS01 (a free base) [37], bafilomycin (a specific vacuolar ATPase inhibitor), and KH7 (a selective soluble adenylyl cyclase inhibitor) [38]. We showed that 100 μM of chloroquine enhanced Tat-mediated LTR transactivation at Tat concentration as low as 0.5 μg/ml (35 nM) (Additional file 1: Figure S1). In the present study, we chose higher concentrations of Tat at 2 μg/ml (140 nM) to induce robust LTR transactivation so that we can demonstrate confidently whether ApoE or blocking LRP1 could affect Tat-mediated LTR transactivation. Using the above conditions (Tat at 2 μg/ml in the presence of 100 μM chloroquine), we next determined the mechanisms by which HIV-1 Tat enters the cells and the involvement of LRP1 in the induction of HIV-1 LTR transactivation in part because LRP1 is involved with HIV-1 Tat internalization [27]. First, using a specific LRP1 antagonist, receptor-associated protein (RAP), we demonstrated that RAP concentration dependently and significantly restricted Tat-mediated HIV-1 LTR transactivation (Fig. 1a). Next, we determined the extent to which siRNA knockdown of LRP1 affected Tat-mediated HIV-1 LTR transactivation. LRP1 protein expression levels were significantly decreased in cells transfected with LRP1 specific siRNA as compared to control (scrambled) siRNA (Fig. 1b). Further, the ability of HIV-1 Tat in conjunction with chloroquine to increase HIV-1 LTR transactivation was significantly attenuated in cells transfected with LRP1 specific siRNA as compared to control (scrambled) siRNA (Fig. 1c). Thus, siRNA knockdown of LRP1 blocked significantly [39] Tat-mediated LTR transactivation. Together, these data indicate that LRP1 is involved in Tat-mediated LTR transactivation in this system.

### ApoE isoform dependently decreased Tat-mediated HIV-1 LTR transactivation in the presence of HDL

**Fig. 2:**
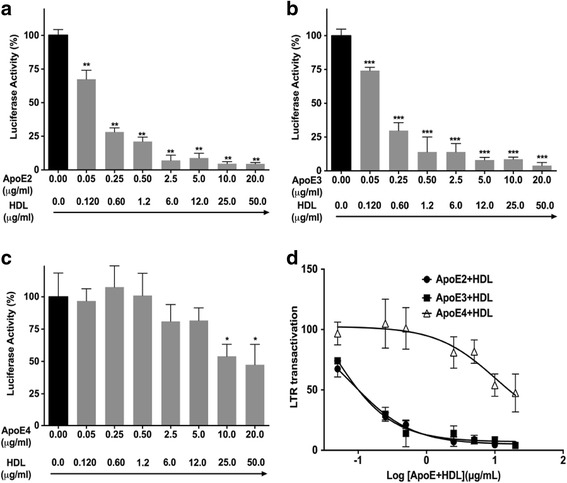
ApoE-HDL isoform dependently affected Tat-mediated HIV-1 LTR transactivation. U87MG cells stably transfected with a luciferase gene under the control of HIV-1 Tat responsive LTR promoter were incubated for 48 h with ApoE2-HDL (**a**), ApoE3-HDL (**b**), or ApoE4-HDL (**c**) in the presence of HIV-1 Tat protein (2 μg/ml) and 100 μM chloroquine (CQ). ApoE2-HDL, ApoE3-HDL, and ApoE4-HDL significantly and concentration dependently restricted Tat-mediated HIV-1 LTR transactivation (*n* = 3; *p < 0.05, **p < 0.01, ***p < 0.001) (**d**). In comparison to ApoE2-HDL and ApoE3-HDL, ApoE4-HDL was less potent and effective at restricting Tat-mediated HIV-1 transactivation

ApoE-rich lipoprotein is the major carrier for cholesterol transport in the brain and is a well-characterized ligand of LRP1. To determine the extent to which ApoE-rich lipoprotein affects Tat-mediated HIV-1 LTR transactivation, we needed to mimic the in situ structure and composition of ApoE-rich lipoproteins in the brain. ApoE-rich lipoproteins synthesized in situ in the brain is thought to be HDL-like particles composed of phospholipids and un-esterified cholesterol [39–41]. We first determined the extent to which plasma-derived HDL (which contains low levels of ApoE) or ApoE-deprived HDL3 affects Tat-induced LTR transactivation, and we found neither plasma-derived HDL nor ApoE-deprived HDL3 at the concentrations of 50 μg/ml affected Tat-induced LTR transactivation (data not shown). To model the brain in situ ApoE-rich lipoproteins, we pre-incubated ApoE2, ApoE3, or ApoE4 with ApoE-deprived HDL3 with a fixed ratio of 0.4 for ApoE protein/HDL protein to form ApoE2-HDL, ApoE3-HDL, and ApoE4-HDL. This ratio was based on the findings that the phospholipid content of human plasma HDL is about 29% and the protein content of human plasma HDL is about 40%; thus, the ratio of phospholipids/protein content of HDL is about 0.7 [42]. Furthermore, the ApoE/phospholipid ratio in CSF is about 0.6 [43]. Therefore, if one assumes that the phospholipid (HDL) content is similar in plasma and CSF, the calculated ratio of ApoE protein/HDL protein content in CSF is 0.42. Using this 0.4 ratio, we demonstrated that ApoE2-HDL (Fig. 2a) and ApoE3-HDL (Fig. 2b), significantly and concentration dependently restricted Tat-mediated HIV-1 LTR transactivation. In contrast, ApoE4-HDL only restricted Tat-mediated HIV-1 LTR transactivation at the two highest concentrations of tested (Fig. 2c). ApoE4-HDL was less potent and less effective than were ApoE2-HDL and ApoE3-HDL at restricting HIV-1 Tat-mediated HIV-1 LTR transactivation (Fig. 2d); calculated IC_50_ values were 0.08 μg/ml for ApoE2-HDL, 0.03 μg/ml for ApoE3-HDL, and 10.0 μg/ml for ApoE4-HDL. Because ApoE2, ApoE3, and ApoE4 were all purified recombinant proteins and may contain endotoxin, the presence of endotoxin in these recombinant proteins may affect Tat-induced HIV-1 LTR transactivation. Thus, we further determined the extent to which endotoxin affects HIV-1 LTR transactivation. We found that endotoxin did not have any effect on Tat-mediated HIV-1 LTR transactivation in U87MG cells (Additional file 1: Figure S3).

### ApoE isoform dependently decreased Tat-mediated HIV-1 LTR transactivation in the absence of HDL

**Fig. 3:**
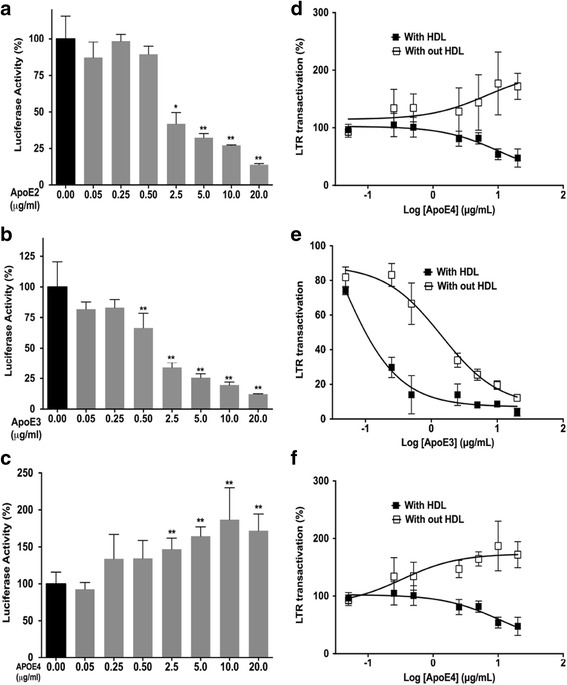
HDL affects the ability of ApoE to restrict Tat-mediated HIV-1 LTR transactivation. **a** ApoE2 significantly and concentration dependently restricted Tat-mediated HIV-1 LTR transactivation in the absence of HDL (*n* = 3; *p < 0.05; **p < 0.01). **b** ApoE3 significantly and concentration dependently restricted Tat-mediated HIV-1 LTR transactivation in the absence of HDL (*n* = 3; **p < 0.01). **c** ApoE4 concentration dependently enhanced Tat-mediated HIV-1 LTR transactivation in the absence of HDL (*n* = 3, **p < 0.01). **d** In the presence of HDL, ApoE2 was more potent and effective at restricting Tat-mediated HIV-1 LTR transactivation. **e** In the presence of HDL, ApoE3 was more potent and effective at restricting Tat-mediated HIV-1 LTR transactivation. **f** In presence of HDL, ApoE4 concentration dependently restricted Tat-mediated HIV-1 LTR transactivation; however, in the absence of HDL, ApoE4 concentration dependently enhanced Tat-mediated HIV-1 LTR transactivation

We have shown that ApoE isoform dependently affected Tat-mediated HIV-1 LTR transactivation in the presence of HDL and that HDL alone does not affect Tat-mediated HIV-1 LTR transactivation. Although we did not determine the extent to which ApoE-HDL affects HIV-1 infection, our finding seems contradict with a previous report that non-lipidated ApoE4 at 10 μg/ml enhances HIV-1 infection in vitro [12]. Thus, we determined the extent to which ApoE isoforms in the absence of HDL-affected Tat-mediated HIV-1 LTR transactivation. We demonstrated that, in the absence of HDL, ApoE2 (Fig. 3a) and ApoE3 (Fig. 3b), but not ApoE4 (Fig. 3c) concentration dependently restricted Tat-mediated HIV-1 LTR transactivation. By comparing results of the effects of ApoEs on Tat-mediated HIV-1 LTR transactivation in the absence or presence of HDL, it was clear that ApoE2 (Fig. 3d), ApoE3 (Fig. 3e), and ApoE4 (Fig. 3f) were all more potent and effective at restricting Tat-mediated HIV-1 LTR transactivation when tested in the presence of HDL (a fixed ratio of 0.4 for ApoE protein/HDL protein); IC_50_ values for ApoE2 were 0.08 μg/ml in the presence of HDL and 2.2 μg/ml in the absence of HDL, and for ApoE3 were 0.03 μg/ml in the presence of HDL and 1.4 μg/ml in the absence of HDL. In the presence of HDL, ApoE4 at the two highest concentrations slightly restricted Tat-mediated HIV-1 LTR transactivation; however, ApoE4 concentration dependently enhanced Tat-mediated HIV-1 LTR transactivation in the absence of HDL (Fig. 3f). Thus, our finding is consistent with the previous report that non-lipidated ApoE4 enhances HIV-1 infection [12].

### ApoE isoform dependently restricted HIV-1 tat internalization

**Fig. 4:**
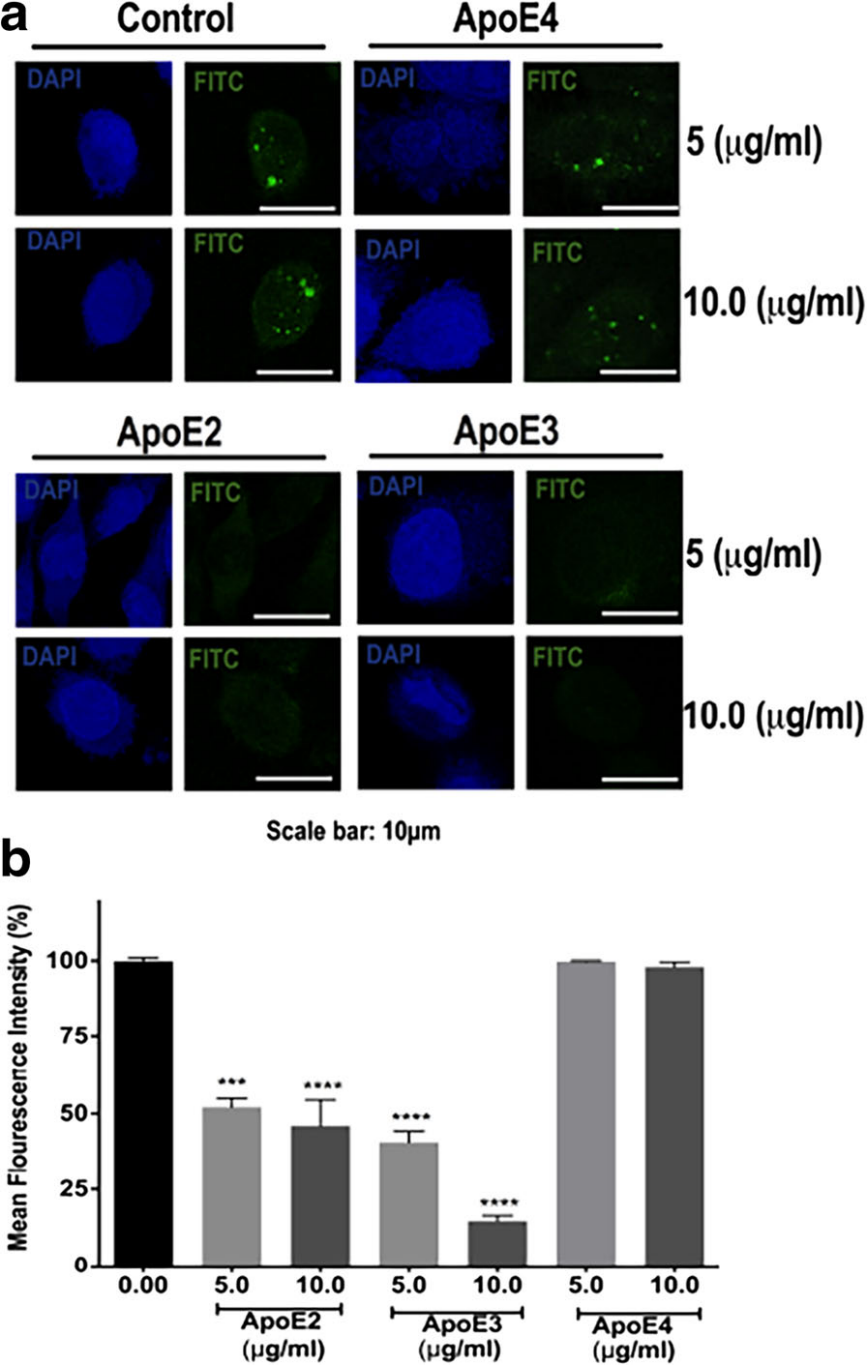
ApoE2 and ApoE3, but not ApoE4, inhibited HIV-1 Tat internalization. Representative images (**a**) and quantified internalized fluorescence intensities (**b**) show that ApoE2 and ApoE3 at 5 and 10 μg/ml inhibited HIV-1 Tat internalization as evidenced by lower levels of punctate staining of internalized FITC-labeled Tat (*n* = 30; ***p < 0.001, ****p < 0.0001), whereas ApoE4 did not inhibit HIV-1 Tat internalization

ApoE and HIV-1 Tat are competitive ligands for LRP1, and ApoE can restrict HIV-1 Tat internalization (26). Accordingly, we next determined the extent to which ApoE2, ApoE3, and ApoE4 affected internalization of HIV-1 Tat. Control cells (Fig. 4a) and cells treated with ApoE4 at 5 and 10 μg/ml (Fig. 4a) exhibited a typical punctate staining pattern for internalized FITC-labeled HIV-1 Tat. ApoE2 at 5 and 10 μg/ml (Fig. 4a), and ApoE3 at 5 and 10 μg/ml (Fig. 4a) displayed less punctate staining of internalized FITC-labeled HIV-1 Tat as compared to control cells. When fluorescence intensity was quantified, FITC-labeled HIV-1 Tat internalization was decreased significantly and concentration dependently for ApoE2 and ApoE3 (Fig. 4b). In contrast, ApoE4 was ineffective in blocking Tat internalization (Fig. 4b).

### Effects of ApoE mimetic peptide and ApoE4 structure corrector on Tat-mediated HIV-1 LTR transactivation

**Fig. 5:**
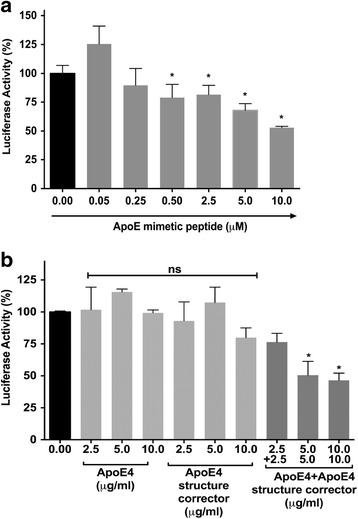
ApoE mimetic peptide decreased Tat-mediated HIV-1 LTR transactivation. **a** U87MG cells were treated with an ApoE mimetic peptide in the presence of HIV-1 Tat and chloroquine (CQ) for 48 h. ApoE mimetic peptide significantly reduced Tat-mediated HIV-1 LTR transactivation (*n* = 3; *p < 0.05). **b** Incubation with an ApoE4 structure corrector, that makes the structure and function of ApoE4 more like ApoE3, enhanced the ability of ApoE4 to restrict Tat-mediated LTR transactivation (*n* = 3; *p < 0.05)

ApoE, a protein with 191 amino acids, has a receptor-binding region at residues 134–150 [44], and biological functions of ApoE are maintained with small fragments consisting of residues 141–150 [45]. We next determined the extent to which an ApoE mimetic peptide (residues 131–169) could affect Tat-mediated HIV-1 LTR transactivation. The ApoE mimetic peptide concentration dependently restricted Tat-mediated HIV-1 LTR transactivation (Fig. 5a). Small-molecule structure correctors (ApoE4 structure correctors) have been shown to modify the structure of ApoE4 to be more of a ApoE3-like phenotype [46]. Using the ApoE4 structure corrector at concentrations ranging from 2.5 to 10 μg/ml, we found that the ApoE4 structure corrector significantly and concentration dependently enhanced the efficiency of ApoE4 in restricting Tat-mediated LTR transactivation (Fig. 5b).

## Discussion

Of the three known human isoforms of ApoE, ApoE4 enhances HIV-1 infection and accelerates HIV-1 disease progression [12, 13, 28]. HIV-1 Tat is an HIV-1 viral protein that is essential for HIV-1 replication. HIV-1 Tat is also a ligand for low-density lipoprotein receptor-related protein 1 (LRP1) and as such can compete for LRP1 binding with ApoEs and can control ApoE-cholesterol uptake. Accordingly, we determined the extent to which different isoforms of ApoE affected Tat-mediated HIV-1 LTR transactivation. Using U87MG glioblastoma cells expressing LTR-driven luciferase, the principal finding of our studies were (1) that a specific LRP1 antagonist, receptor-associated protein, and siRNA knockdown of LRP1 both restricted significantly Tat-mediated LTR transactivation, (2) that of the three ApoEs, ApoE4 was the least potent and effective at preventing HIV-1 Tat internalization and decreasing Tat-mediated HIV-1 LTR transactivation, (3) that Tat-mediated LTR transactivation was attenuated by an ApoE mimetic peptide, and (4) that ApoE4-induced restriction of Tat-mediated LTR transactivation was potentiated by an ApoE4 structure modulator that changes ApoE4 into an ApoE3-like phenotype.

Resting memory CD4+ T cells in the blood are the best characterized cellular HIV-1 reservoirs [47]. However, there are other sites of HIV persistence in the body. The brain is unique in terms of its “immune privileged” status, and the presence of the blood-brain barrier limits efficacious access to anti-retroviral drugs, and evidence does suggest that the brain is a viral reservoir for HIV-1 [48, 49]. Indeed, HIV-1 enters the brain within the first week of systemic infection and targets perivascular macrophages, microglia, and astrocytes [16, 17], and HIV DNA has been detected in the brains of HIV+ individuals whose infection was controlled by combined anti-retroviral therapy [50], and partial integrated HIV-1 provirus has been detected in the brain cells [50–53]. In the brain, macrophages, microglia, and astrocytes can be infected by HIV-1. However, HIV-1 infection in macrophages or microglia is often productive while infection in astrocytes is less productive [54]. It is not clear what causes such a nuance. It is reported that endocytosis is involved in HIV-1 entry in both macrophages [54, 55] and astrocytes [54], and different set of receptors are used for HIV-1 entry in macrophages or astrocytes, which could partially account for the restrictive nature of HIV-1 infection in astrocytes. It has been reported that in HIV-1-infected brain, up to 19% astrocytes carry HIV-1 DNA [56, 57] and because of their abundance astrocytes could be an important viral reservoir in brain. The existence of such viral reservoirs that act as sanctuaries for HIV-1 have made the complete eradication of HIV-1 extremely challenging [50, 51, 57]. The HIV-1 protein Tat represents an attractive target for HIV-1 eradication of HIV-1 because HIV-1 Tat is essential for viral transcription [21–23] and because brain levels of Tat remains elevated even though HIV-1 virus is below detectable levels [58]. HIV-1 Tat is secreted from HIV-1 infected cells and can bind to cell surface receptors including LRP1, heparin sulfate proteoglycan, CD26, and CXCR4. HIV-1 Tat enters all CNS cells via receptor-mediated endocytosis [25, 27, 31, 59] and following its internalization into endolysosomes, HIV-1 Tat enters the nucleus and activates the HIV-1 LTR promoter [26, 60, 61]. The importance of HIV-1 Tat to HIV-1 replication is further highlighted by findings that in the absence of HIV-1 Tat, HIV-1 LTR transcription terminates prematurely due to inefficient elongation [21–23].

Currently, local concentrations of Tat in HIV-1-infected tissues are not known. In Tat-transfected cell lines, nanomolar concentrations of Tat were detected in HIV-1-infected cells [61], sera of HIV-1-infected individuals [62, 63], and in CSF of HIV-1-infected individuals [58]. However, even those levels are likely to underestimate Tat concentrations in HIV-1-infected tissues [64] because Tat is easily denatured and oxidized [65, 66], and it can be sequestered by endogenous anti-Tat antibodies [67, 68] and/or by glycosaminoglycans [63]. Tat protein is present in the brain tissue, as evidenced by the demonstration of Tat reactivity in post-mortem brain tissue of HIV+ individuals [58] and in HIV-infected macaques [27]. Such brain Tat could be secreted from infiltrating T cells and mononuclear cells [58] and infected perivascular microphage, microglia cell, and astrocytes [16, 17]. While in situ brain levels of Tat are unknown, nanomolar concentrations of Tat were detected in CSF of HIV-infected individuals [58]. Tat was detected in the CSF of only three of eight HIV-infected individuals [58], and this may be due to the non-uniformity of HIV-1 infection, and the fact that protein levels in CSF are much lower compared to protein levels in brain interstitial fluid. Even though Tat may not be measurable in some cases [69], it is clearly present because it is essential for viral replication [21–23, 26, 60, 61] and Tat transcripts are produced earlier and in greater quantities in infected cells than are p24 or gp41 [70, 71]. This should not be viewed as unusual because Tat has yet to be measured in human brain or for that matter in the brains of Tat-transgenic mice. In addition, as shown for the well-accepted neurotoxins glutamate and kainate, alterations in the size of the extracellular space may change what might be a physiological concentration to a pathological concentration [72]. The size of the extracellular space varies from 10 to 20% in different brain regions and is dynamic; significant shrinkage of the space occurs with glial cell swelling and hypertrophy, as well as with changes in pH, potassium, and sodium. Thus, Tat levels in HIV-1-infected brain may vary dramatically depending on the factors listed above as well as Tat’s physical-chemical properties. Furthermore, Tat could accumulate in the brain over time even in ART-treated individuals. In support, protease inhibitors such as Darunavir do not prevent the formation of Tat in vitro [58], and it has been shown that antiviral therapy does not block the secretion of Tat in HIV-infected individuals [67]. Thus, it is not unreasonable for us to speculate that in situ brain levels of Tat could reach 2 μg/ml (140 nM) level as used in the present study, especially in local brain regions around HIV-1-infected cells.

Because Tat is easily denatured and oxidized [65, 66], higher (nM to μM) concentrations of Tat are often required when treating cells with Tat for prolonged periods. Typically, robust HIV-1 LTR transactivation requires high concentrations of exogenous HIV-1 Tat. In COS cells transiently transfected with HIV-1 LTR chloramphenicol acetyl transferase (CAT) reporter, Tat at concentrations greater than 0.5 μg/ml induced LTR transactivation [61], whereas in HL3T1 cells stably integrated with HIV-1 LTR CAT reporter and TZM-bl cells stably integrated with HIV-1 LTR-β-galactosidase reporter, Tat at concentrations of up to 1 μg/ml did not induce LTR transactivation [32]. In our system where U87MG was stably integrated with HIV-1 LTR luciferase reporter, Tat at concentrations of up to 8 μg/ml did not induce LTR transactivation. However, chloroquine, a well-known anti-malarial drug that de-acidifies endolysosomes significantly, enhances Tat-mediated HIV-1 LTR transactivation [26, 31–33]. Consistent with these findings, we found that the optimal concentration of chloroquine to markedly enhance Tat-mediated HIV-1 LTR transactivation was 100 μM. We showed here that 100 μM of chloroquine enhanced Tat-mediated LTR transactivation at Tat concentration as low as 0.5 μg/ml (35 nM). In the present study, we chose higher concentrations of Tat at 2 μg/ml (140 nM) to induce robust LTR transactivation so that we can demonstrate confidently whether ApoE or blocking LRP1 could inhibit Tat-mediated LTR transactivation. Currently, the underlying mechanism whereby chloroquine enhances Tat-induced LTR transactivation is not known. It has been speculated that chloroquine-induced de-acidification of endolysosomes decreases Tat catabolism and enhances the nuclear delivery of Tat [32]. Consistent with this idea, lipofectamine-based transfection reagents, which recently have been shown to avoid lysosome degradation [73], has the ability to enhance exogenous Tat-induced LTR transactivation [31, 32]. Although chloroquine is not present in the brain, many other factors in HIV-1 infected brain could act similarly as chloroquine to induce endolysosome de-acidification and enhance Tat-mediated LTR transactivation. Given that gp120 and TNFα have both been shown to neutralize endolysosome pH [34, 36], we determined the extent to which gp120 and TNFα affected Tat-mediated LTR transactivation in the absence of chloroquine, but neither affected Tat-mediated LTR transactivation. However, we demonstrated that other endolysosome de-acidifying reagents act in a similar way as chloroquine to enhance Tat-mediated LTR transactivation, and these reagents include a known free base (LYS01) [37], a specific vacuolar ATPase inhibitor (bafilomycin), and a selective soluble adenylyl cyclase inhibitor (KH7) [38].

It is well known that Tat has the extraordinary ability to cross plasma membrane transporting a variety of macromolecules into a plethora of cell types. Tat can enter all CNS cells via endocytosis following interactions with specific cell surface proteins and receptors [25, 27, 31, 59]. Following endocytosis and internalization into endolysosomes, Tat can either harbor in endolysosomes or escape into the cytosol, where it transits to the nucleus and activates the HIV-1 LTR promoter [26, 60, 61]. It remains an open question on how Tat escapes endolysosomes. Interestingly, chloroquine has been shown to dramatically enhance Tat endolysosome escape [74]. However, the concept that chloroquine neutralizes endolysosome pH and prevents degradation of Tat in endolysosomes only partially explains why chloroquine enhances Tat endolysosome escape. Further mechanistic studies are warranted.

LRP1 is a large type I transmembrane protein, composed of a large 515-kDa N-terminal extracellular subunit and a non-covalently associated 85-kDa C- terminal transmembrane subunit [75]. The extracellular domain has four ligand-binding domains I–IV with 2, 8, 10, and 11 cysteine-rich complement-type repeats, respectively. The LRP1 ligand-binding domains II and IV are the major LRP1 binding regions interacting with a diverse array of approximately 40 structurally diverse ligands including ApoE [76]. The binding of ApoE to LRP1 leads to its endocytosis [77]. It is important to note that the core domain of Tat (amino acids 37–48) is directly involved in Tat interaction with LRP1 domains II, III, and IV [27]. Thus, ApoE could compete with Tat to bind LRP1. Using a stably transfected cell line with HIV-1 LTR-driven luciferase, we determined ApoE isoform-dependent effects on HIV-1 LTR transactivation. Tat-mediated HIV-1 LTR transactivation in U87MG cells was clearly mediated by LRP1 because the transactivation was decreased significantly by pharmacological inhibition using receptor-associated protein and by a siRNA knockdown strategy. In the absence of HDL, ApoE2 and ApoE3 concentration dependently blocked HIV-1 Tat internalization and attenuated Tat-mediated HIV-1 LTR transactivation; however, ApoE4 in the absence of HDL was inefficient in blocking HIV-1 Tat internalization and enhanced Tat-mediated HIV-1 LTR transactivation. Such a finding is consistent with previous report that non-lipidated ApoE4 at 10 μg/ml enhances HIV-1 infection in vitro [12]. Moreover, HDL binding to the ApoEs clearly increased the potency and effectiveness of all three ApoE isoforms in restricting Tat-mediated HIV-1 LTR transactivation. In the presence of HDL, the IC_50_ values for ApoE2 and ApoE3 to restrict Tat-induced HIV-1 LTR transactivation are 80 and 30 ng/ml, respectively. These IC_50_ values are close to reported ApoE concentrations (46 ng/ml for ApoE2 and 18 ng/ml for ApoE3) in mouse brain interstitial fluid [39], but much lower than reported human CSF ApoE concentrations (2.69–8 μg/ml) [43, 78]. Thus, we feel confident that our findings suggest that the brain in situ HDL-like ApoE lipoproteins exert restrictive effects on Tat-induced LTR transactivation.

The observed isoform-dependent effects of ApoE on Tat-mediated HIV-1 LTR transactivation could be due to the differences in their ability to bind lipids and receptors. Amino acid differences among the three isoforms of ApoE are restricted to residues 112 and 158 [1], and the change from cysteine to arginine affects their structure and their ability to bind lipids and receptors [4, 5]. Previous reports have shown that non-lipidated ApoE does bind to LRP1, but with much less affinity compared to lipdated ApoE [79–82]. In addition, both structure and functional analyses have shown that ApoE3, but not ApoE4, preferentially binds and transports HDL, and the binding of HDL lipids opens the hinge region between N- and C-terminal domain of ApoEs allowing receptor-binding domain free access to ApoE receptors [6, 83, 84]. Indeed, ApoE4 has been shown to be less effective than ApoE2 or ApoE3 in binding LRP1 [82]. Our findings suggest that ApoE4 is less efficient than ApoE2 or ApoE3 in competing with Tat for LRP1 binding, blocking HIV-1 Tat internalization, and blocking Tat-mediated LTR transactivation.

The N-terminal of ApoE contains the putative receptor-binding site (residues 141–150) for LRP1 [44], and ApoE mimetic peptides containing the receptor-binding region exhibit biological functions of ApoE [45]. Here, we demonstrated that ApoE mimetic peptide attenuated Tat-mediated HIV-1 LTR transactivation. Thus, ApoE mimetic peptide might be used as a therapeutic strategy against HIV-1. Furthermore, because small-molecule structure correctors (ApoE4 structure correctors) modify ApoE4 structure to be more ApoE3-like [46], we tested the effects of an ApoE4 structure corrector and demonstrated that the ApoE4 structure corrector enhanced the efficiency of ApoE4 in restricting Tat-mediated HIV-1 LTR transactivation.

## Conclusions

Our findings indicate that ApoE4 is less effective than ApoE2 or ApoE3 in restricting Tat-induced HIV-1 LTR transactivation in glioblastoma, ApoE mimetic peptide could be used as a therapeutic strategy against HIV-1 infection and associated neurocognitive disorders, and ApoE4 structure correctors could be used as a therapeutic strategy in HIV-1 positive people having the APOE4 allele. Future studies investigating ApoE4 structure correctors and their ability to affect HIV-1 infection in astrocytes and macrophage/microglia cells and disease progression are warranted.

## Additional file

Additional file 1: **Figure S1.** Chloroquine enhances Tat-mediated LTR transactivation in U87MG cells. **Figure S2.** Endolysosome de-acidifying reagents enhance HIV-1 Tat-mediated LTR transactivation in U87MG cells. **Figure S3.** Endotoxin (LPS) does not affect Tat-mediated HIV-1 Tat transactivation in U87MG cells. (DOCX 1981kb)
