# Supplementary material for: Apolipoprotein E isoform dependently affects Tat-mediated HIV-1 LTR transactivation
Source: J Neuroinflammation. 2018 Mar 20;15:91. doi: 10.1186/s12974-018-1129-1 (PMC5861635; doi:10.1186/s12974-018-1129-1)
Supplement: Supplementary file 1 — Figure S1. Chloroquine enhances Tat-mediated LTR transactivation in U87MG cells. Figure S2. Endolysosome de-acidifying reagents enhance HIV-1 Tat-mediated LTR transactivation in U87MG cells. Figure S3. Endotoxin (LPS) does not affect Tat-mediated HIV-1 Tat transactivation in U87MG cells. (DOCX 1981kb) [file 12974_2018_1129_MOESM1_ESM.docx]

**Additional file**

**(A)**

**(B)**


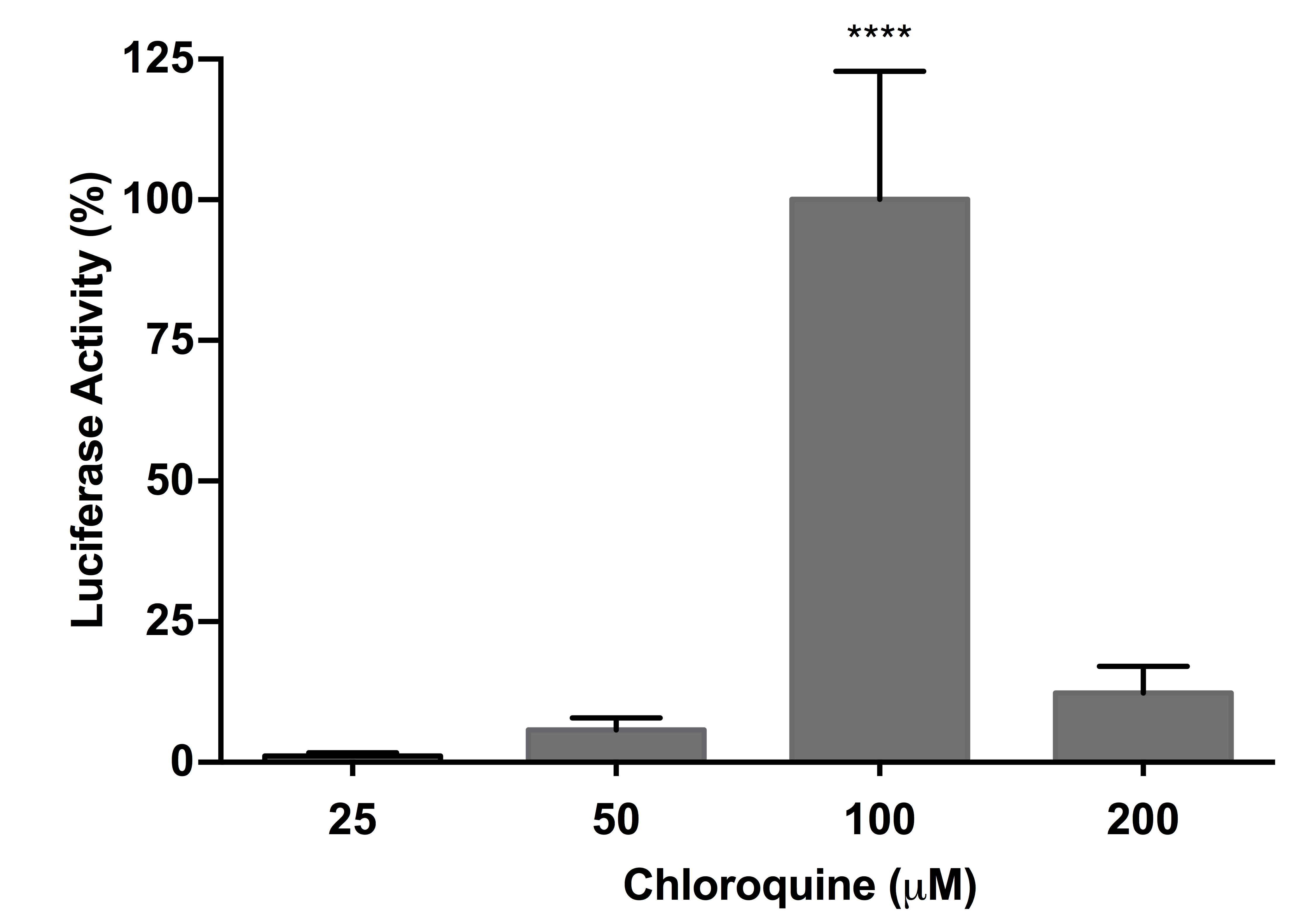

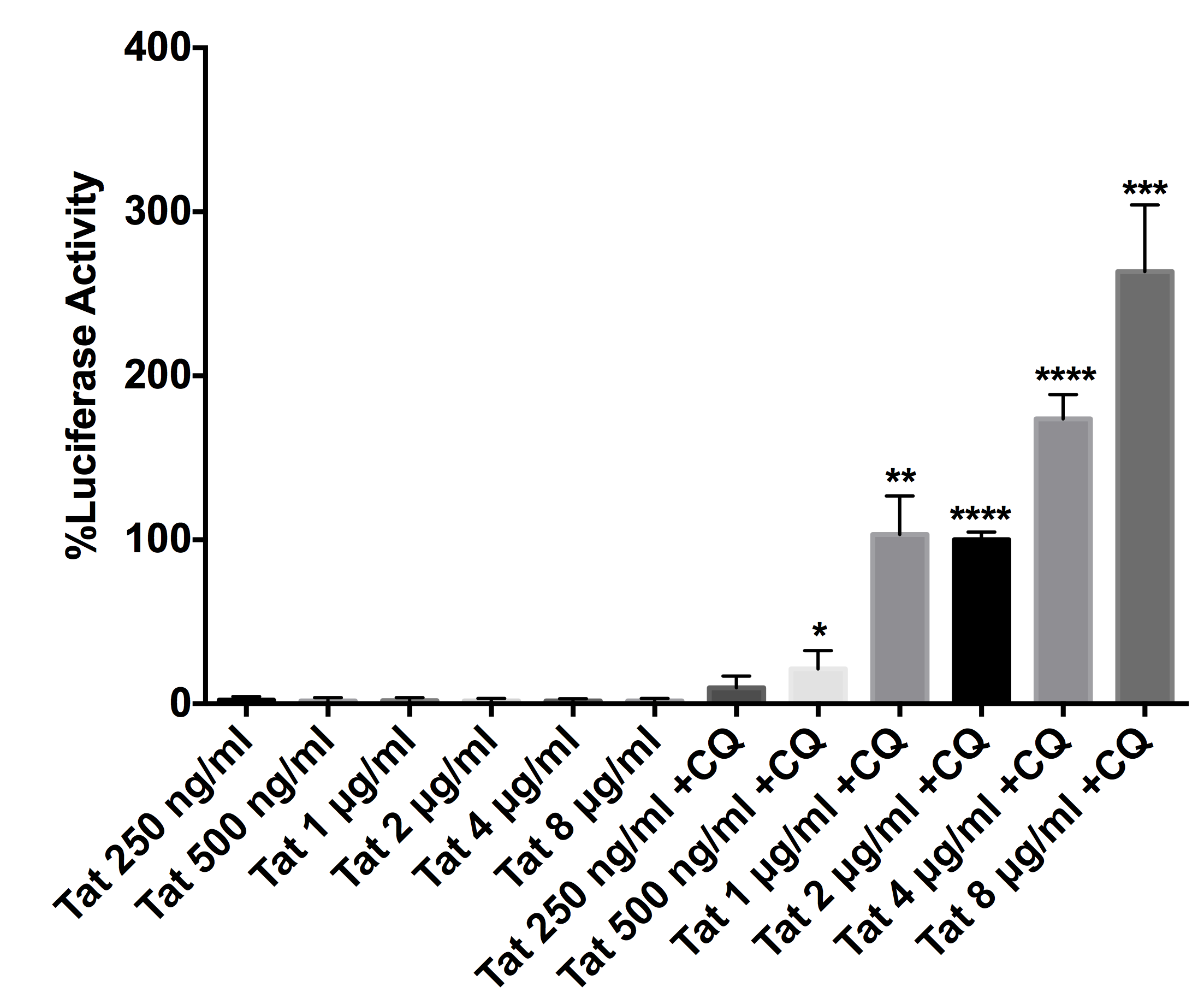


**Figure S1. Chloroquine enhances Tat-mediated LTR transactivation in U87MG cells. (A)** U87MG cells were treated with HIV-1 Tat protein at doses ranging from 250 ng/ml to 8 μg/ml in the absence or presence of chloroquine (100μM) for 4hrs. In the absence of chloroquine, HIV-1 Tat did not induce measurable levels of HIV-1 LTR transactivation. **(B)** U87MG cells were treated with HIV-1 Tat protein (2 μg/ml) in the presence of chloroquine at concentrations ranging from 25 to 200 μM. HIV-1 LTR transactivation measured as relative luminescence units was enhanced optimally at a chloroquine concentration of 100 μM. (n=3; **p<0.01; ***p<0.001)


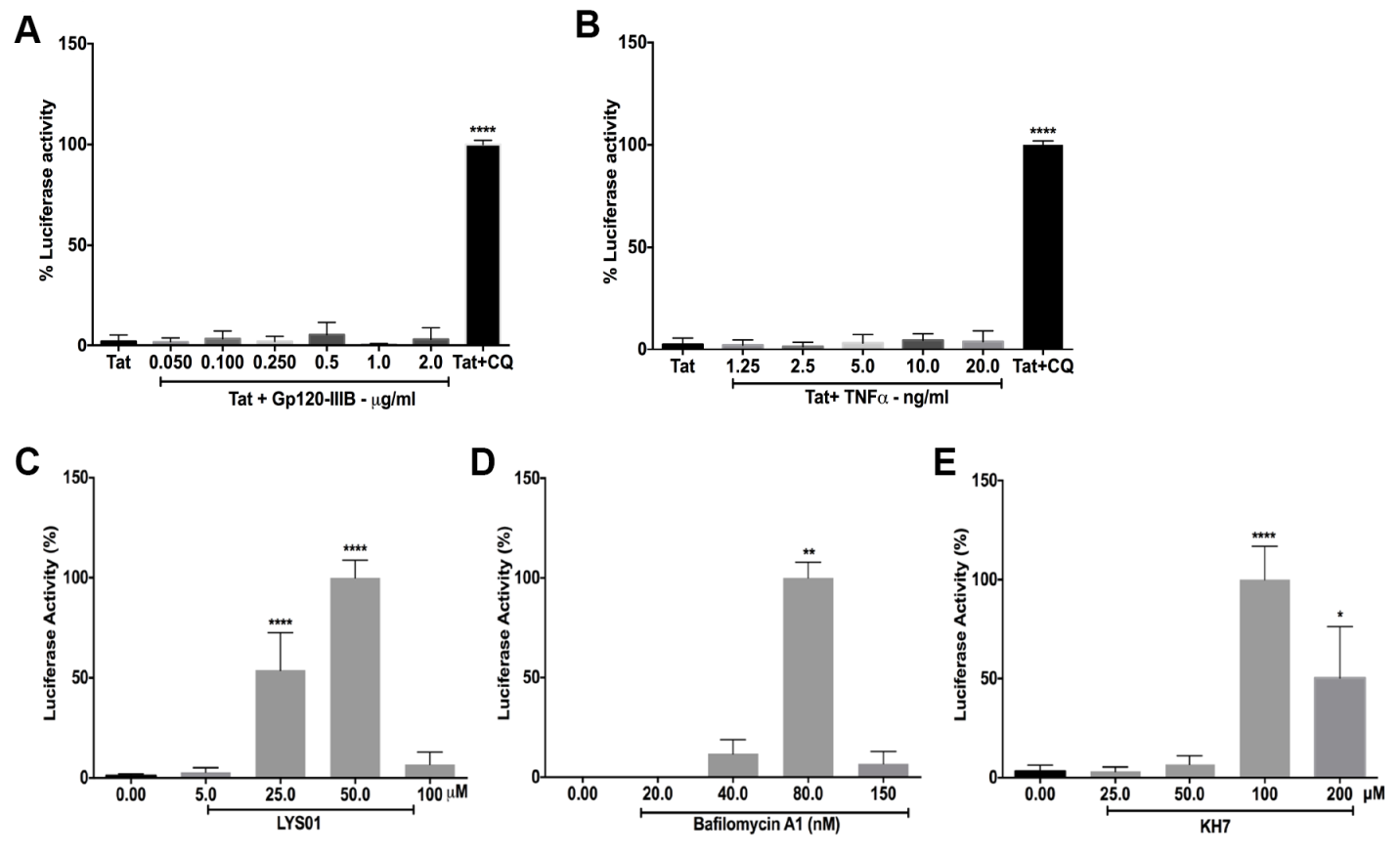


**Figure S2.** **Endolysosome de-acidifying reagents enhance HIV-1 Tat-mediated LTR transactivation in U87MG cells**. (**A)** U87MG cells were treated with HIV-1 Tat protein (2 μg/ml) in the presence of gp120 at concentrations ranging from 0.05 to 2 μg/ml. HIV-1 LTR transactivation measured as relative luminescence units was not affected by gp120. (**B)** U87MG cells were treated with HIV-1 Tat protein (2 μg/ml) in the presence of TNFα at concentrations ranging from 1.25 to 20 ng/ml. HIV-1 LTR transactivation measured as relative luminescence units was not affected by TNFα. **(C)** U87MG cells were treated with HIV-1 Tat protein (2 μg/ml) in the presence of LYS01 (a free base) at concentrations ranging from 5 to 100 μM. HIV-1 LTR transactivation measured as relative luminescence units was enhanced optimally at a LYS01 concentration of 50 μM. (n=3; ****p<0.0001). **(D)** U87MG cells were treated with HIV-1 Tat protein (2 μg/ml) in the presence of bafilomycin (a specific vacuolar ATPase inhibitor) at concentrations ranging from 20 to 150 nM. HIV-1 LTR transactivation measured as relative luminescence units was enhanced optimally at a bafilomycin concentration of 80 nM. (n=3; **p<0.01). **(E)** U87MG cells were treated with HIV-1 Tat protein (2 μg/ml) in the presence of KH7 (a selective soluble adenylyl cyclase inhibitor) at concentrations ranging from 25 to 200 μM. HIV-1 LTR transactivation measured as relative luminescence units was enhanced optimally at a KH7 concentration of 100 μM (n=3; *p<0.05; ****p<0.0001).

**Figure S3. Endotoxin (LPS) does not affect Tat-mediated HIV-1 Tat transactivation in U87MG cells.** U87MG cells were treated with HIV-1 Tat protein (2 μg/ml) with chloroquine in the presence of endotoxin (Millipore; LPS25- 0111:B4) at concentrations ranging from 1 to 10 ng/ml. Endotoxin did not affect Tat-induced HIV-1 LTR transactivation. (n=3; ns, p>0.05)
